# A World Health Organization Human Hepatitis E Virus Reference Strain Related to Similar Strains Isolated from Rabbits

**DOI:** 10.1128/genomeA.00292-18

**Published:** 2018-04-19

**Authors:** Marco Kaiser, Déborah Delaune, Olivier Chazouillères, Johannes Blümel, Anne-Marie Roque-Afonso, Sally A. Baylis

**Affiliations:** a GenExpress Gesellschaft für Proteindesign mbH, Berlin, Germany; b Virologie, AP-HP Hôpital Paul Brousse, Villejuif, France; c Hépatologie, AP-HP Hôpital Saint Antoine, Paris, France; d Paul-Ehrlich-Institut, Langen, Germany

## Abstract

We report here the genome sequence of a hepatitis E virus (HEV) strain from a chronically infected immunodeficient patient. Full-length sequence analysis revealed a distinct HEV strain, of a tentative new subgenotype, clustering with viruses from rabbits. It is a World Health Organization reference strain for validation of nucleic acid testing.

## GENOME ANNOUNCEMENT

Although first identified in people, certain strains of hepatitis E virus (HEV) have been found to be widespread in animals such as domestic pigs and wild boar. Ingestion of meat and meat products derived from such animals is responsible for the large increase in autochthonous cases of hepatitis E reported in Europe and other developed regions in recent years (1). Chronic infections can occur in immunosuppressed patients infected with a zoonotic HEV strain, particularly one that belongs to genotype 3 (2).

Strains of HEV have also been identified in wild rabbits (3–5), captive rabbits, including household pets and those in petting farms (4, 6), farmed rabbits (3, 7, 8), and laboratory animals (9, 10). HEV strains from rabbits have been identified in China (7, 10), South Korea (8), North America (11), France (3), Italy (6), Germany (5), and the Netherlands (4). Recently, strains of HEV were identified in hares (5). Although most HEV genotype 3 strains identified in people are related to swine viruses, a very small number of cases of hepatitis E have been observed in immunocompromised patients that cluster with rabbit HEV strains in a distinct clade (3, 12).

An international reference panel (IRP, code number 8578/13) of HEV strains has been prepared on behalf of the World Health Organization (WHO) for validating nucleic acid tests and standardizing assays (13, 14). A human HEV strain related to viruses found in rabbits is included in the WHO IRP and was obtained from a stool sample from a 47-year-old French male who presented with elevated levels of alanine aminotransferase while undergoing routine surveillance 9 years after receiving a liver transplant as a result of hepatitis B virus-related cirrhosis. Sequence analysis of the HEV strain was performed as previously described (15).

Phylogenetic analysis demonstrated that the strain from the patient was most closely related to rabbit strains of HEV. The patient strain grouped most closely with HEV strains detected in rabbits from France (GenBank accession numbers JQ013791 and JQ013792) and Germany (GenBank accession number KY436898). Nevertheless, nucleotide identity was only ∼81 to 83% based on the full-length HEV sequences, thus confirming that the patient strain represented a tentative new subgenotype. Like strains of HEV from rabbits, there was a 93-bp nucleotide insertion in the X domain of ORF1.

Infection caused by the HEV strain reported here is one of only a small number of cases, all in immunocompromised individuals who have been shown to have been infected with a strain of HEV related to viruses reported to date found in rabbits (3, 12). The patient was a restaurant worker who regularly handled pork and rabbit meat and may have been infected with rabbit HEV by a knife injury. Knowledge of the sequence of this strain is important because this has been included in the WHO IRP (13), and the strain was not always well detected by participating laboratories. It is important to ensure that assays are adequately designed to detect with certainty strains that might infect humans; this is particularly important for immunocompromised patients, for whom serological screening is unhelpful.

### Accession number(s).

The sequence of the HEV strain reported here has been deposited in GenBank under the accession number MG211750. The related sequence from the strain derived from serum from the patient was deposited in GenBank under the accession number MG211751.
